# *In vitro* Models for Seizure-Liability Testing Using Induced Pluripotent Stem Cells

**DOI:** 10.3389/fnins.2018.00590

**Published:** 2018-08-31

**Authors:** Alastair I. Grainger, Marianne C. King, David A. Nagel, H. Rheinallt Parri, Michael D. Coleman, Eric J. Hill

**Affiliations:** Life and Health Sciences, Aston University, Birmingham, United Kingdom

**Keywords:** iPSC neurons, astrocytes, seizures, *in vitro*, safety pharmacology

## Abstract

The brain is the most complex organ in the body, controlling our highest functions, as well as regulating myriad processes which incorporate the entire physiological system. The effects of prospective therapeutic entities on the brain and central nervous system (CNS) may potentially cause significant injury, hence, CNS toxicity testing forms part of the “core battery” of safety pharmacology studies. Drug-induced seizure is a major reason for compound attrition during drug development. Currently, the rat *ex vivo* hippocampal slice assay is the standard option for seizure-liability studies, followed by primary rodent cultures. These models can respond to diverse agents and predict seizure outcome, yet controversy over the relevance, efficacy, and cost of these animal-based methods has led to interest in the development of human-derived models. Existing platforms often utilize rodents, and so lack human receptors and other drug targets, which may produce misleading data, with difficulties in inter-species extrapolation. Current electrophysiological approaches are typically used in a low-throughput capacity and network function may be overlooked. Human-derived induced pluripotent stem cells (iPSCs) are a promising avenue for neurotoxicity testing, increasingly utilized in drug screening and disease modeling. Furthermore, the combination of iPSC-derived models with functional techniques such as multi-electrode array (MEA) analysis can provide information on neuronal network function, with increased sensitivity to neurotoxic effects which disrupt different pathways. The use of an *in vitro* human iPSC-derived neural model for neurotoxicity studies, combined with high-throughput techniques such as MEA recordings, could be a suitable addition to existing pre-clinical seizure-liability testing strategies.

## Seizures

The brain is vulnerable to damage by physical trauma and a multitude of injurious agents, including pathogens, pharmaceuticals, and toxins. Physiological and anatomical protection and regulation for the controlled environment of the brain is provided by the blood–brain barrier (BBB). This consists of brain endothelia, astrocytes, and pericytes, providing a system of tight endothelial junctions where selective permeability to water, some gases, and fuel sources such as glucose and amino acids can be modulated (Daneman and Prat, 2015). This separation of the brain from the systemic blood circulation maintains comprehensive and minute control of its biochemistry, especially via glial cells which regulate the ionic and nutrient composition of fluid surrounding neurons (Prat et al., 2001). Should these mechanisms fail, the risk of brain injury is increased. Indeed, seizure is one such severe neurological complication that can present either as a result of an adverse drug reaction (ADR), infection, or from trauma (Vaughan and Delanty, 2002; Koseki et al., 2014).

A seizure is the defining symptom of epilepsy, which is one of the most common chronic neurological disorders, estimated to affect 65 million individuals worldwide (Thurman et al., 2011). “Epilepsy” encompasses multiple syndromes which predispose the individual to generation of epileptic seizures (Fisher et al., 2005). A seizure itself is defined as an abnormal, transient discharge of neurons in the brain (Fisher et al., 2005), and is broadly characterized by neuronal hyperexcitability and hypersynchrony (Jiruska et al., 2013). If an individual suffers a single event with no recurrence, they are said to have suffered a *seizure*. If multiple consecutive and/or recurring seizures are experienced, the patient may be diagnosed with *epilepsy* (Scharfman, 2007). Furthermore, “epileptogenesis” describes the processes which render a healthy system capable of generating seizures, whilst also establishing the condition, hence making recurrent seizures more likely (Pitkänen, 2010). Patients suffering from seizures experience different effects depending on the brain region involved. These may include changes in cognition, paresthesia, or the experience of flashing lights or unusual odors. Convulsions are commonly observed, which can be accompanied by various combinations of muscle rigidity (tonic) and jerking limb (clonic) activity.

Although seizure is the defining symptom of the epilepsies, only about 25% of patients who suffer seizure have an epilepsy syndrome (Stasiukyniene et al., 2009). The remaining patients suffer seizures from the major causes listed above, as well as neonatal occurrences among infants. These seizures may be described as *provoked* or *acute symptomatic*, as they are not the result of established or enduring brain alterations, but rather occur in an acute and transient manner (Fisher et al., 2005; Thurman et al., 2011). The potential severity of seizures makes them an important metric for neurotoxicity assays.

## What Do We Expect From *In Vitro* Models of Seizure-Liability Testing?

**FIGURE 1:**
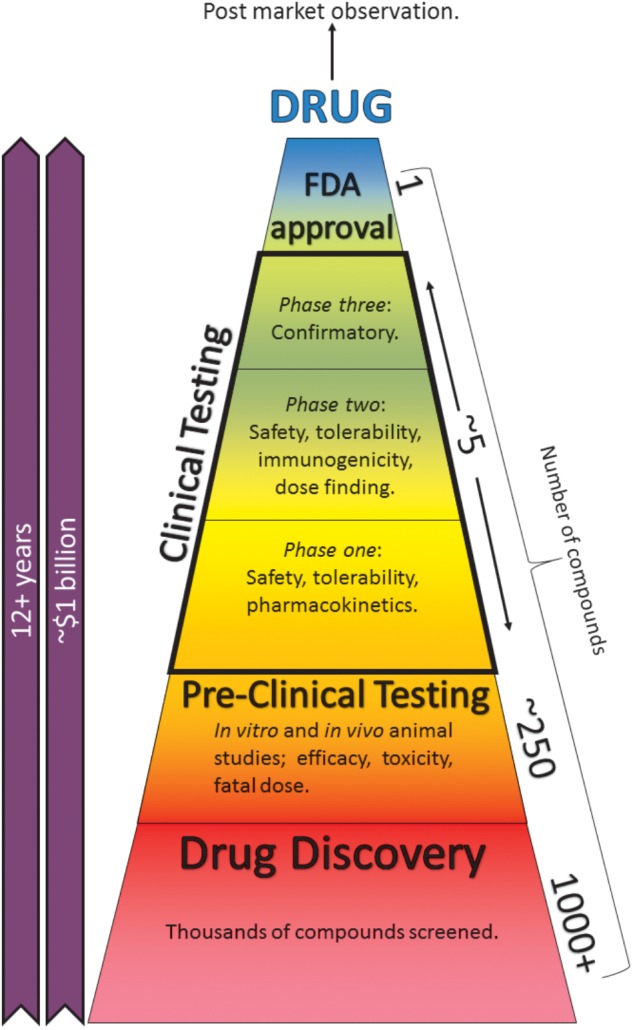
The drug-development process. Taking anywhere up to 15 years and a cost of ∼$1.7 billion, the process can be separated into pre-clinical and clinical testing. Pre-clinical studies consist of *in silico*, *in vitro*, and *in vivo* animal and cell-based assays. Post-market surveillance continues indefinitely (Mundae and Östör, 2010). Reducing these studies or finding better alternatives can save time, cost, and resources.

Central nervous system (CNS) toxicity testing of any new pharmaceutical is a vital procedure and legal requirement for safety pharmacology studies (ICH, 2000). A drug-induced seizure is an example of a potentially fatal ADR and is the most commonly encountered CNS-related issue during the drug development process (Authier et al., 2016). Pre-clinical seizure-liability (PSL) testing is essential to identify such ADRs; however, these tests usually occur late in the drug development process (**Figure 1**; Easter et al., 2007). Despite the severity of drug-induced seizure, there are no official guidelines outlining how this issue should be tested and regulated (Easter et al., 2009). *In vitro* models enable potential side effects to be discovered earlier, thereby saving time, cost, and resources. However, current models are hindered by limitations such as low-throughput capabilities, arguable relevance to man and a heavy reliance on animal studies, which often require considerable expense. Furthermore, *in vivo* PSL testing frequently requires specialist practitioners, also likely to increase costs and decrease throughput.

An ideal model system for *in vitro* PSL testing should be able to recapitulate the information existing models provide and address some of their limitations. The most obvious shortcoming for current platforms is that they use animals. An ideal platform would therefore be relevant to humans, through the use of cells which are anatomically human, expressing human receptor proteins with which current convulsant and anti-convulsant compounds can interact. The cells should also be able to form functional neurophysiological networks, containing the various cell types seen in the intact brain. Importantly, the platform must be capable of pharmacological interrogation, of displaying phenotypic human seizure-like activity and of sensitivity to known therapeutic anti-convulsants. The system should also be robust and amenable to high-throughput testing and predictive of the effects of diverse neuroactive compounds.

Current effective *in vitro* models include rodent brain tissue slices and CNS cell cultures. These have been employed for decades and are credited with the discovery of mechanisms pertinent to seizure, epilepsy, and neurobiology in general. Animal-based models of seizure are important for defining epileptiform and seizure activity, creating standards and descriptions to which the field can relate their findings. While “seizure” describes a full seizure event *in vivo*, the term “seizure-like event” (SLE) more accurately describes the effects seen in *in vitro* and predictive *in silico* models. A human-based *in vitro* seizure-liability platform should fundamentally be capable of generating human SLEs.

## Current *In Vitro* Models of Seizure

There are many models for seizure-liability testing to choose from. For simplicity, the following section will focus on the models most commonly used in toxicity screening and a comparison of these models is shown in **Table 1**.

**Table 1:** Comparison of current major seizure-liability testing models with proposed iPSC-derived model.

Model	Benefits	Limitations
*Acute slice assay*	• Representative of *in vivo* rodent adult brain • Same day experimentation • Validated for use • Defined cytoarchitecture • Current ‘gold standard’ • Forms functional network	• Difficulty in inter-species extrapolation • Preparations undergo cellular changes and damage • Projection neurons severed • Typically low throughput
*Organotypic slice culture*	• Representative of *in vivo* rodent • Retain connective properties of the tissue • Can recover from damage from slicing • Can assess long-term effects of neuroactive chemicals • Forms functional network	• Difficulty in inter-species extrapolation • Derived from neonates, so may not be predictive of matured system • More time consuming • Requires supportive culture medium • Synaptic reorganization/remodeling
*Primary CNS culture*	• Representative of cell subtypes *in vivo* • Predictive model validated • Higher throughput than slices	• Difficulty in inter-species extrapolation • Loss of structure and 3-dimensionality • More time consuming • Do cultured cells reach maturity? • Often cultured in absence of astrocytes or other neural cells
*iPSC-derived culture*	• Human-based • Exhibit humanoid morphology • Retain genotype of original fibroblast, so can be used to model genetic components of epilepsy from patients with specific mutations • Amenable to increased throughput • No ethical considerations	• Expensive • Time consuming • Research still in infancy, lacking validation • No defined ultrastructure • No guarantee of presence of desired cell types • No standard protocol

### Acute Slice Assays

In a 2016 industrial survey of nervous system safety pharmacology, the rat *ex vivo* hippocampal slice (HS) assay was found to be most commonly used for seizure-liability testing (Authier et al., 2016). Slices can be obtained from any region of any species with a complex brain. HSs make for useful *in vitro* models as they retain a defined cytoarchitecture and relevant receptors and constituents of the full brain system, including inter-area connectivity. This is of great importance, as the cause of seizures and epileptiform activity can be complex and interlinked with multiple hierarchical levels of the CNS (Scharfman, 2007). External conditions can be precisely controlled and manipulated with HSs. Indeed, slice assays typically involve manipulation of the extracellular ionic milieu to induce SLE, as discussed in Section “Inducing Seizure-Like Events Using *in vitro* Models.” Furthermore, the mechanisms by which different agents induce SLE is highly variable. With tissue slices, there is a greater likelihood that all necessary cell types and receptors to respond to diverse pharmacological agents are present. Acute slices are harvested from adult rodent brain, intended for experimentation on the same day to study individual neurons or neuronal circuits (Lein et al., 2011). However, difficulties in inter-species extrapolation reduce the utility of the platforms and inevitably, slice preparations undergo important cellular and environmental changes including ischemia and severing of projection neurons that would have connected the slice to the rest of the system. Obviously, human brain tissue is problematic to obtain (except for limited excised epileptic tissue), so efforts to refine the commonly used rodent HS or replace with non-human primates have been attempted (Easter et al., 2007). Once removed from the animal, slices rapidly begin to deteriorate, making them an expensive and low-throughput model system; however, a semi-automated platform has been validated for use in pre-clinical testing, allowing multiple slices per animal to be perfused simultaneously, and hence, increasing the throughput capabilities of the platform (Easter et al., 2007).

### Organotypic Slice Assays

In contrast to acute slice preparations, organotypic slices (OS) are derived from neonatal rodents and are maintained *in vitro* for several weeks (Lein et al., 2011). Compared with acute slices, OS require more time and resources, particularly a culture medium which can provide essential growth factors, hormones and metabolites; which may or may not include serum. As variability between culture media can frequently be attributed to serum, chemically defined media have also been developed and have been shown to support OS culture and more importantly facilitate seizurogenesis (Liu et al., 2017).

Organotypic slices are representative of their respective *in vivo* counterpart. They contain most of the neuronal subtypes present in the brain and retain intrinsic connective properties of the tissue. As previously mentioned, excision of acute slices leads to cellular damage, ischemia, and an altered metabolic state; however, OS can recover from these insults during culture. Furthermore, any necrotic cells or debris disappears after several weeks in culture (Lein et al., 2011). OS are particularly useful for assessing long-term effects of agents or SLEs, as they can be further incubated following experimentation.

Despite the supposed benefits of OS, there are limitations in using them as models, *cf* acute slices. There is concern that as the tissue is harvested from neonates, this is not entirely representative of adult tissue. While any acute damage to OS rectifies itself, synaptic reorganization and axonal/dendritic remodeling can occur because of deafferentation (Gutiérrez and Heinemann, 1999). The trauma of slicing can also activate glial cells, leading to the formation of an astrocytic scar, which was believed to influence the prevention of axon regeneration, although now this is disputed (Lein et al., 2011; Anderson et al., 2016).

### Primary Cell Culture Assays

The second most commonly used model system for seizure-liability studies is primary neuronal cell cultures (Authier et al., 2016). Similarly to slice models, primary rodent CNS cultures can contain most, if not all, of the components of intact cortex, which bear a true resemblance to the cells *in vivo*. However, the structure and three-dimensional nature of the brain is lost. Unlike slice assays, cell cultures are typically higher throughput, but do take several weeks in culture to reach maturity, as is the case with OS.

While neurons are an absolute requirement for seizure activity, they are not the only cell type involved. Indeed, omitting other cell types, or culturing ratios of subtypes not representative of *in vivo* ratios could affect the validity of such models. There is also concern that certain receptors or channels may not appear in cultured neurons, which may or may not influence the outcome of experiments (Dichter and Pollard, 2006). In spite of this, primary cell culture assays can reliably and consistently predict seizure liability. Bradley et al. (2018) have recently published a comprehensive *in vitro* screen for seizure liability using primary rat cortical neurons, including ratios of neurons and astrocytes observed in the intact rat brain (Bradley et al., 2018). In addition, Kreir et al. (2018) have also illustrated the predictive capability of rat cortical models from cultures containing both excitatory and inhibitory neuronal subtypes (Kreir et al., 2018). These rodent cells can respond to a large group of agents known to induce SLEs, with consideration of multiple neural activity markers (Bradley et al., 2018; Kreir et al., 2018). However, like OS, there is controversy over when the cultured cells reach maturity, and at which point they should be used for seizure-liability studies so that they are as representative of the living system as possible. Arguably, the most important consideration for epileptiform studies is the capability of cells to evolve network functionality, which can develop, propagate, and sustain SLEs.

### Astrocytes and Their Role in Seizure

Many studies of seizures describe properties of neuronal cells and networks. However, glial cells such as: astrocytes, oligodendrocytes, microglia, and Schwann cells perform vital support tasks in the nervous system. Within the CNS, numbers of glial cells have been overestimated in the past, and it has been shown that the neuron:glia ratio varies throughout areas of the brain (von Bartheld et al., 2016). In the cerebellum, glial cells make up only 18.9% of cells, while in the rest of brain, this figure increases to 91.7% (Herculano-Houzel, 2014). Despite this significant quantity, it is only relatively recently that astrocytes have been studied in sufficient depth to appreciate the full diversity of their roles in the brain.

Excessive glutamatergic excitation of neurons can cause excitotoxicity, resulting in damage to, and the eventual death of the neuron (Maragakis et al., 2004). Astrocytes take up excess ammonia and glutamate from the synaptic cleft via the GLT1 (EAAT2) transporter (Maragakis et al., 2004) and use glutamine synthetase to convert it to glutamine via a condensation reaction (Choi, 1987). Inhibition of astrocytic glutamine synthetase has been implicated in multiple neurodegenerative disorders, including epilepsy (Eid et al., 2013). Furthermore, GLT1 knockout rats have an increase in neuronal cell death and in extracellular glutamate concentration, characteristic of excitotoxicity (Rothstein et al., 1996).

In addition to glutamate, healthy astrocytes also balance potassium levels. During AP discharge, extracellular K^+^ increases, with the concentration increasing considerably during seizure (Feldberg and Sherwood, 1957). Accumulation of extracellular K^+^ would lead to the resting membrane becoming more positive, affecting gating of ion channels, transporters, and various receptors. Astrocyte membranes are highly permeable to potassium with many potassium channels along with sodium/potassium ATPases, and so prevent against such events (Carmignoto and Haydon, 2012). In rodent models, these transporters are responsible for maintaining low extracellular K^+^ levels and importantly, restore resting levels of K^+^ following epileptiform activity (Coulter and Steinhauser, 2015). Dysfunction to astrocytic uptake of extracellular K^+^ and astrocytic uncoupling can lead to the generation and propagation of seizure (Bedner et al., 2015).

### Human Tissue

Although the focus of this review is on models for drug-induced seizures, research using resected human tissue from patients with drug-resistant epilepsy should be considered. The possibilities for human tissue range from histopathological analyses, to modulation of SLE in drug-resistant tissue and the testing of novel anti-convulsant compounds and electrophysiological investigation (Gabriel et al., 2004; Hsiao et al., 2015; Jones et al., 2016; Klaft et al., 2016). Furthermore, a number of methods are now available that increase the longevity of the resected tissue, enabling increased throughput of investigations (Schwarz et al., 2017; Wickham et al., 2018). However, it is unlikely that a sufficient amount of human tissue will ever be available for high-throughput compound screening. Furthermore, the tissue is often in a diseased state, which may demonstrate different responses to otherwise healthy tissue. Ethical considerations and consent also need to be obtained to use human tissue, further complicating an already limited process. Human stem cells could provide a viable alternative to human tissue, allowing researchers to generate functional neuronal networks for high-throughput drug testing.

## iPSCs

Induced pluripotent stem cell (iPSC) technology (Takahashi and Yamanaka, 2006) has considerable potential for toxicity testing and disease modeling, allowing the generation, growth, and study of human cells without the need for invasive isolation procedures or extensive ethical approval. The application of iPSCs extends from neurotoxicity testing and disease modeling to drug-screening and cell-based therapies (Jung et al., 2012; Kumar et al., 2012; Pei et al., 2016).

Neuronal cultures which are derived from human iPSC could be a suitable addition to PSL testing, as the systems are closer to humans than a primary rodent-derived cell line. In fact, human iPSCs are very similar to embryonic stem cells (Robinton and Daley, 2012). In addition, a remarkable benefit of iPSCs is that they retain the genotype of the original fibroblast cell and indeed, any cell then generated from iPSCs also shares that genetic background. This is invaluable in disease research, as cells taken from both patients and controls can be studied and compared.

### iPSC-Derived Seizure-Liability Models

**FIGURE 2:**
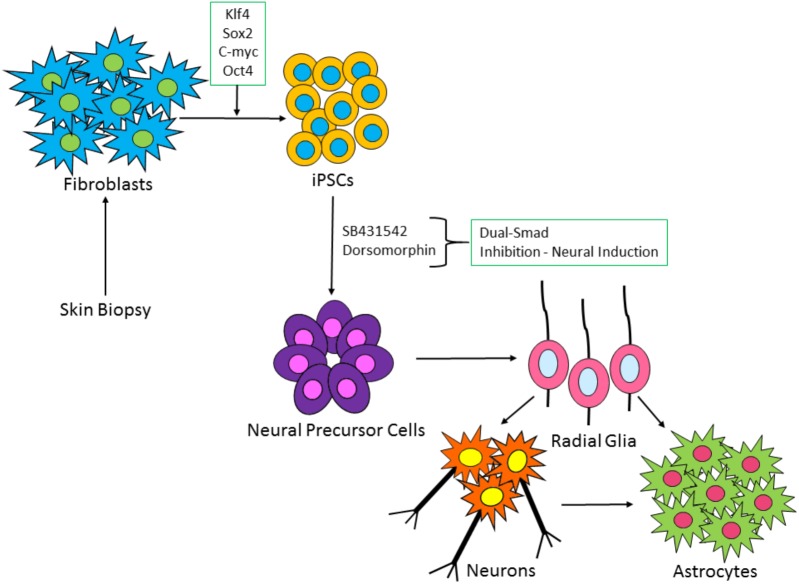
Schematic of iPSC-derived neuronal and glia cell generation from fibroblast cells. Patient fibroblasts are reprogrammed with master regulators of pluripotency: c-Myc, OCT4, Klf-4, and SOX2. iPSC colonies are formed, which via a process known as dual-SMAD inhibition can induce the iPSCs to a neural fate. Following neural precursor cell formation, radial glia-like cells are produced which differentiate over time into neurons and astrocytes.

The foremost aim of *in vitro* neurotoxicity tests, including iPSC-derived PSL models, should be to replicate the *in vivo* morphology and functionality as closely as possible. Diseases of the cerebral cortex are major causes of morbidity and mortality. Hence, iPSC-derived cortical neuronal systems may provide a reliable predictive base for PSL testing and are conceptually more relevant to toxicity testing than animal tissue, as they are human cells. Protocols for the development of iPSC-derived cortical neurons and characterization of cortical neurogenesis and terminal differentiation to achieve mature electrophysiological properties and functional excitatory synapses have been developed, as shown in **Figure 2** (Chambers et al., 2009; Shi et al., 2012a; Gunhanlar et al., 2018). The development of iPSC-derived cortical cultures should demonstrate the presence of cortical neuronal markers, astrocytes, and populations of excitatory and inhibitory neurons, (which exist at roughly 80–20% in the human cortex, respectively). Indeed, the cortical induction method proposed by Shi et al. (2012b) showed the generation of astrocytes following spontaneous differentiation. Moreover, it has been shown that human iPSCs can also be differentiated exclusively into astrocytes (Shaltouki et al., 2013) and interneurons (Liu et al., 2013; Kim et al., 2014). This is of critical importance when considering the seizurogenic potential of novel compounds. An entirely excitatory or inhibitory culture is not a representative system and true responses to novel compounds may not be accurately observed without inclusion of both major neural subtypes. Indeed, Tukker et al. (2018) have reported that known seizurogenic agents may not display typical reponses in cultures of iPSC-derived neurons and astrocytes, where there is an unnatural ratio of excitatory to inhibitory neurons (Tukker et al., 2018). In addition, significant differences in drug responses have been reported between different iPSC-derived neuronal cells, highlighting the importance of mixed cultures (Pei et al., 2016). Efforts have been made to determine the presence of excitatory and inhibitory neuronal populations and neuron/astrocyte ratios and it would appear that immunocytochemical staining for morphological assessment and pharmacological interrogation are valid methods to suggest the presence/absence of such neuronal subtypes and astrocytes (Kuijlaars et al., 2016; Tukker et al., 2016; Gunhanlar et al., 2018; Tukker et al., 2018). While excitatory and inhibitory iPSC-derived neural cells and iPSC-derived astrocytes can now be commercially obtained, the key to a healthy responsive system is the balance (and presence) of these specific subtypes in a given culture. A complete, demonstrative, heterogenous system must include all subtypes and at ratios close to those observed *in vivo*.

### iPSC-Derived Astrocytes in a Cortical Model of Seizure Liability

Neurons cannot function *in vivo* without astrocytes. Among their many functions, astrocytes are highly involved in the formation and maturation of excitatory and inhibitory synapses in the CNS, and also their degradation and removal to refine neural circuits (Nguyen et al., 2011). For example, rodent retinal ganglion cells form very few synapses when cultured alone, but the number is increased 10-fold in the presence of astrocytes or astrocyte-conditioned media (ACM; Pfrieger and Barres, 1997).

Several studies using iPSC-derived neural models have co-cultured human iPSC-derived neurons together with rodent astrocytes or ACM (Tang et al., 2013; Odawara et al., 2014, 2016a,b). The differences between rodent and human astrocytes are quite considerable, ranging from their size and function to the presence of morphologically distinct astrocyte subtypes within the human brain and the cortex in particular (Oberheim et al., 2009). However, studies using iPSC neurons and rodent astrocytes have highlighted the importance of including astrocytes in culture. Human-neuronal and rodent-astrocytic co-cultures demonstrate enhanced functional maturation, spiking activity, and maintenance of long-term electrical activity *cf* neurons alone (Odawara et al., 2014; Lischka et al., 2018). It has recently been suggested that co-culture of human neurons with rodent astrocytes may generate matured spontaneous electrical activity to a greater extent than using human astrocytes (Lischka et al., 2018). However, this may reflect the relative maturity of iPSC- derived astrocytes used in these studies.

Studies of stem cell-derived astrocytes have demonstrated that these cells are capable of exhibiting many of the functions of human *in vivo* astrocytes, including: facilitating synaptic maturation, gliotransmission, protection of neurons from excitotoxicity and stress, and astrocytic calcium activity (Hill et al., 2016). An increasing number of studies have seeded iPSC-derived neurons with human iPSC-derived astrocytes in short-term cultures (Tukker et al., 2016, 2018; Kayama et al., 2018; Matsuda et al., 2018) and with a specific focus on neurotoxicity testing and seizure-liability testing (Ishii et al., 2017; Tukker et al., 2018). It has been demonstrated that iPSC-derived neural co-cultures are capable of generating epileptiform activity in response to convulsants (Ishii et al., 2017), and that network activity, bursting activity, and synchronicity is facilitated by the inclusion of human iPSC-derived astrocytes (Tukker et al., 2018). However, a full characterization and validation of seizure-liability, similar to the acute slice work of Easter et al. (2007), is yet to be performed using iPSC-derived systems. Furthermore, assessing the effects of compounds on astrocytes will provide increased accuracy and validity, particularly as astrocyte-specific agents can be tested and monitored in mixed cultures (Hill et al., 2012).

Co-culturing iPSC-derived neuronal cells with astrocytes ensures the development of a heterogenous neural model. The inclusion of astrocytes is important, as astrocyte dysfunction may be involved in seizure activity, as discussed in Section “Astrocytes and Their Role in Seizure.” However, there are a variety of ways to generate these cultures. During *in vivo* brain development, astrocyte differentiation follows radial glia and neuronal differentiation (**Figure 2**). To our knowledge, no study has attempted to generate co-cultures following this spontaneous differentiation protocol, for the express purposes of seizure-liability testing. It would be of great interest to determine any differences in the predictivity of cultures generated by seeding neuronal cells and astrocytes together (Kuijlaars et al., 2016; Tukker et al., 2016, 2018; Ishii et al., 2017; Kayama et al., 2018, Matsuda et al., 2018), compared to spontaneous differentiation (Shi et al., 2012a,b; Kirwan et al., 2015; Kuijlaars et al., 2016; Gunhanlar et al., 2018; Paavilainen et al., 2018), wherein mixed populations of neurons and astrocytes are generated. The spontaneous route is not as rapid, with emergence of astrocytes seen around 6 weeks in culture and establishment of electrical activity around 9–15 weeks (Shi et al., 2012b; Kirwan et al., 2015; Paavilainen et al., 2018). As the field progresses, it will be necessary to determine an acceptable time frame for development of these cultures, without sacrificing maturation and predictive capabilities of convulsant drug effects.

## Functional Analysis of iPSC-Derived Cultures

While there are now many emerging disease platforms and investigative models using iPSC-derived neural cells, research using human iPSC neural networks for the express purposes of drug screening is still in its infancy. It has been shown that iPSC-derived neurons respond appropriately to convulsants, pro-convulsants, and conditions known to generate epileptiform activity and as such, could be utilized in PSL testing (Odawara et al., 2014, 2016b; Tukker et al., 2016, 2018; Ishii et al., 2017; Kayama et al., 2018; Paavilainen et al., 2018). While typical endpoints for current PSL tests include assessment of biochemical, morphological and physiological endpoints, the throughput capabilities of these assays are too low for toxicity screening. Changes to ion channels, calcium changes, and network responses are implicated in many toxicity pathways and functional techniques used must be able to detect these changes. Convulsant compounds affect the nervous system and neuronal excitability as discussed in Section “Inducing Seizure-Like Events Using *in vitro* Models.” These disruptions in turn affect nervous system physiology, often preceeding or occuring in the absence of the typical morphological/biochemical changes. Furthermore, some existing methods fail to record the most rapid events, such as action potentials, or are not amenable to high-throughput testing, making them less desirable for toxicity screening. Therefore, methods used with iPSC-derived neural models must demonstate a relatively high-throughput capability, without compromising on the quality of the information obained. Techniques enabling network-wide effects to be recorded and visualized would provide relevant endpoints to monitor drug induced SLEs.

In many cases, techniques such as patch clamping are employed to monitor electrical activity and responses; however, these are invasive and are traditionally limited to measurement of a single cell. Patch clamping is also a very technically demanding and precise method and requires a high level of expertise. However, recent developments have enabled multiple-cell patch clamping to be used in certain industrial applications (Vardi et al., 2016). While patch clamping is a particularly efficient and accurate method of studying ionic currents, its use for large-scale toxicity screening is not practical.

### Calcium Imaging

Fluorescent calcium imaging is a well-established technique which enables visualization of free intracellular Ca^2+^ from populations of cells. Calcium indicators are sensitive to calcium movement and can be loaded in a non-invasive manner to neuronal cells, although prolonged exposure to the dye is toxic. Fluorescent dyes can either be single or multiple wavelength. Calcium imaging enables the researcher to explore the role calcium is playing in the cell, due to specific calcium-mediated processes occuring over different time periods. For example, calcium-mediated neurotransmitter release occurs much more rapidly than calcium-mediated gene expression in the nucleus (Grienberger and Konnerth, 2012). With regard to PSL, calcium imaging can be used to detect electrical activity, oscillatory activity, synchrony, and network activity, making it a very useful tool for assessing neural circuitry and drug responses (Smetters et al., 1999). This approach has been used in an effort to characterize the development and maturation of iPSC-derived neural calcium activity and to study network responses to neurotransmitters/agents (Kirwan et al., 2015; Tukker et al., 2016; Paavilainen et al., 2018). These studies have demonstrated maturation of oscillatory calcium activity and appropriate responses to a panel of agents. However, to the authors knowledge, the use of calcium imaging for PSL testing in iPSC-derived neural networks has not yet been demonstrated. In contrast, the response of primary neural cultures to a comprehensive panel of neurotransmitters and convulsants has been shown by multi-well calcium imaging (Pacico and Mingorance-Le Meur, 2014).

Recent advancements in the development of genetically encoded calcium indicators (GECIs) removes the need for fluorescent dye loading. GECIs, like their traditional counterparts, are non-invasive, but can be targeted to specific neurons (or astrocytes), allowing longer duration imaging, without the risk of photo-toxicity (Mank and Griesbeck, 2008). Other issues observed with fluorescent dyes such as background fluorescence and non-specific dye loading can be overcome with GECI technology (Mank and Griesbeck, 2008). Combining GECI with iPSC-derived models could provide a new dimension to PSL and arguably increase the throughput and efficiency of this technique.

### Multi-Electrode Array Recordings

Multi-electrode array (MEA) systems are increasingly used with iPSC-derived cultures to assess spontaneous electrical activity, synchronous epileptiform bursting activity, drug responses, and network mechanisms, such as long-term potentiation and depression (Odawara et al., 2014, 2016a,b; Frega et al., 2017; Ishii et al., 2017; Seidel et al., 2017; Matsuda et al., 2018; Tukker et al., 2018). MEAs take advantage of the generation of neuronal ion currents. MEAs transduce this ionic voltage change to electronic current, which can then be detected and analyzed by a wide range of commerically available and custom-made software. This technology is non-invasive, enabling the real-time analysis of activity in multiple locations in cultured neurons and also slices. They can record extracellular potentials and basic measures such as spiking and bursting activity and network responses (Johnstone et al., 2010). The use of primary rat cortical neurons on the MEA is well characterized, with extensive neural activity endpoints. Multi-well MEAs have also significantly increased the throughput capabilities of primary cell culture assays. The parameters and results from primary models are important for providing a comparative system for emerging iPSC-derived MEA data (Bradley et al., 2018). A number of studies have been carried out to determine whether MEA analysis of iPSC-derived neural networks is a viable option for toxicity screening. Furthermore, a broad panel of drugs have been evaluated, and results suggest that MEAs are a useful tool in screening compounds with diverse mechanisms of action (McConnell et al., 2012; Valdivia et al., 2014; Kasteel and Westerink, 2017).

As the throughput capabilities of MEAs increase and their usage in toxicity screening becomes more widespread, it will be important to generate standard protocols and definitions of SLEs in iPSC-derived models. Recent studies have assessed a wide variety of endpoints to demonstrate epileptiform activity and network functionality, with primary rodent cells (Bradley et al., 2018) and iPSC-derived neural co-cultures (Tukker et al., 2018). While there are some commonalities between approaches, each group using MEAs for neurotoxicity screening assess different endpoints based upon their respective experimental design. For example, a common endpoint groups consider is the number of spikes in a burst, with bursts being typical of epileptiform activity. Matsuda et al. (2018) have recently published a stepwise method for detection of synchronized burst firings in iPSC-derived neural cultures, which is a positive move toward standard protocols for assessing epileptiform activity using the MEA (Matsuda et al., 2018).

## Culture Medium and Experimental Solution Considerations

The first stage of any project to evolve a reliable protocol for iPSC usage is to optimize the basic conditions for growth and differentiation, as these conditions can influence structural and functional endpoints. Growth media is the most important determinant of these outcomes. Evidently, as the cellular culture itself should be as representative of *in vivo* as possible, all integrated factors such as the media should also aim to mimic *in vivo* compositions. Multiple companies and institutions are now producing their own media, leading to great variation within the field. Many of these media are based on well-established neurobasal and/or DMEM recipes (Shi et al., 2012a; Bardy et al., 2015). Interestingly, it has been shown that concentrations of salts (in particular, NaCl) and the presence of serum may directly influence certain electrical activity (Bardy et al., 2015), which creates another issue for functional imaging studies, as groups are imaging their cells using different fluid compositions.

Artificial cerebrospinal fluid (aCSF) is a mimic of the fluid surrounding the brain *in vivo* and contains the necessary salts and pH to maintain cells and elicit functional recordings. Indeed, aCSF is widely used for slice assays and primary cultures and for inducing and assessing epileptiform activity. Due to the range of medium available for iPSC culture, it may be that a defined aCSF formulation should be used for calcium imaging/MEA studies to create a standard protocol for iPSC-derived neural toxicity testing. It is also possible to obtain human cerebrospinal fluid (hCSF) from patients, which has been shown to have a protective effect on resected human tissue, promoting longevity compared with culture medium (Schwarz et al., 2017). Furthermore, preservation of electrophysiological properties, including network-level activity, is observed. While it appears unlikely that hCSF would be readily available in the quantities necessary to culture and assess iPSC-derived neural models, it would nevertheless be interesting to determine what effects, if any, this composition has on the electrophysiological activity of the cultures.

## Inducing Seizure-Like Events Using *In Vitro* Models

Existing assays used to study SLE manipulate cellular processes in otherwise healthy tissue, in order to initiate seizurogenesis and generate SLE. Seizurogenesis describes the generation of a seizure, falling under the broad umbrella of epileptogenesis – modifications in the brain to support seizure development. It is likely that epileptogenic mechanisms occur before, throughout, and following seizurogenesis to support seizure propagation, leading to changes within the brain which are receptive to seizurogenesis before a seizure occurs (Blauwblomme et al., 2014). However, neither of these processes are entirely understood and both seizurogenesis and epileptogenesis can arise from multiple mechanisms, which adds increased complexity. On the most elementary level, one can consider seizurogenesis to be the result of perturbation to the delicate balance between neuronal excitation and inhibition. Indeed, suppressing inhibition and enhancing excitation are both criteria for epileptic discharges (Lerche et al., 2001).

The induction of SLE is well-established in primary *in vitro* models and brain slice assays, using agents which alter ion flux, neurotransmission and affect the whole electrical network (Yaari et al., 1983, 1986; Pena and Tapia, 2000; Gabriel et al., 2004; Debanne et al., 2006; Easter et al., 2007; Gonzalez-Sulser et al., 2011; Igelstrom et al., 2011; Accardi et al., 2017). In order to generate a SLE, the system must exhibit typical healthy processes and receptors/drug targets for the convulsants to act upon. The following sections briefly describe some of the common methods that have been used to induce SLE. These can also be considered a prerequisite for a valid iPSC-derived seizure-liability model.

### Manipulating Ion Levels

Neuronal excitability is controlled by ion gradients. The manipulation of ion levels in neurons can result in increased activity and SLE. Changes to potassium, calcium, and magnesium levels are typically achieved by modulation of the bathing solution used to perfuse the cell/tissue for the experiment.

Elevated extracellular potassium concentrations ([K^+^]_e_) can lead to depolarization, increased firing, and burst firing, all of which facilitate seizurogenesis. Furthermore, during seizure, [K^+^]_e_ increases and extracellular sodium and calcium ([Na^+^]_e_/[Ca^2+^]_e_) decreases, due to neuronal release and uptake, respectively (Raimondo et al., 2015). This can create a cycle of depolarization, promoting further AP discharge. Increasing potassium levels is an established method for generating SLE *in vitro* (Yaari et al., 1986; Traynelis and Dingledine, 1988). Several studies have demonstrated that iPSC-derived neurons can respond to high potassium solutions, with increases in intracellular calcium observed (Tukker et al., 2016; Paavilainen et al., 2018).

Lowering the extracellular concentration of Ca^2+^ can induce regular SLE, accompanied by transient decreases in Na^+^ and increases in K^+^ in the extracellular space (Yaari et al., 1983). Low [Ca^2+^]_e_ enhances neuronal excitability and can induce spontaneous, synchronized bursts of activity, reminiscent of epileptiform discharges. Calcium influx occurs via voltage gated-calcium channels and *N*-methyl-D-aspartate (NMDA) receptors and during seizure, the [Ca^2+^]_e_ decreases far lower than non-convulsing tissue (Simons, 1988; Somjen, 2002; Blauwblomme et al., 2014). This decrease is due to calcium influx into neurons undergoing the seizure activity, which impairs synaptic transmission as there is too little Ca^2+^_e_ to sustain the calcium influx. Higher Ca^2+^_e_ can enhance synaptic transmission as calcium is available to enter the cell, even though a higher [Ca^2+^]_e_ reduces excitability (as the cell is not depolarized). However, this remains to be observed in iPSC-derived models.

Lowering the concentration of Mg^2+^ can affect the system at the network level, leading to recurrent SLE (Igelstrom et al., 2011; Pacico and Mingorance-Le Meur, 2014). Normal activity is asynchronous, so for seizures to occur, multiple neurons need to be recruited in an unusually hypersynchronous manner (Vaughan and Delanty, 2002; Jiruska et al., 2013). The cerebral cortex is prone to generating synchronous, large bursts of activity which facilitates seizurogenesis (Bromfield et al., 2006). Bursts are generated by sustained recurrent excitation, elicited by clusters of glutamatergic pyramidal neurons in the cortex, and followed by a period of hyperpolarization. Recurrent excitation via NMDA and non-NMDA glutamatergic receptor activation can further recruit and synchronize neurons into the seizurogenic activity (McCormick and Contreras, 2001; Debanne et al., 2006). This sequence of events is termed the paroxysmal depolarizing shift (PDS). Normally, magnesium (Mg^2+^) blocks the pore of the NMDA receptor. During the PDS, the membrane becomes depolarized to the point where the voltage-dependent Mg^2+^ block of NMDA receptors is released. When Mg^2+^ is lowered, NMDA receptors become permeable to Ca^2+^ which, alongside voltage-gated Na^+^ activation, induces long-lasting potentiation of glutamatergic transmission at pyramidal cell synapses (Staley and Dudek, 2006). Activation of multiple NMDA receptors further depolarizes the cell and promotes increased Ca^2+^ influx (Vaughan and Delanty, 2002). Indeed, NMDA and AMPA receptor antagonists are well-known to suppress seizurogenesis (Rogawski, 1992).

### Pharmacological Induction of Seizure-Like Events

Seizure-like events can also be induced pharmacologically in seizure models, with most agents promoting increased bursts of activity from release of excitatory and inhibitory neurotransmitters. Common GABA agonists such as benzodiazepines and barbiturates are known to be anti-convulsive and function by enhancing the inhibitory effect of GABA (Wong, 2010). Similarly, antagonistic agents which block GABA synthesis (such as isoniazid) are documented pro-convulsants (Treiman, 2001).

An agent now used in the therapy of multiple sclerosis, 4-aminopyridine (4-AP), is an experimental compound also widely used to induce SLE and increase neuronal activity in rodent models (Pena and Tapia, 2000; Easter et al., 2007; Gonzalez-Sulser et al., 2011; Hongo et al., 2015; Accardi et al., 2017; Bradley et al., 2018; Kreir et al., 2018) and has been shown to elicit recurrent depolarizations in human iPSC-derived networks (Pruunsild et al., 2017; Matsuda et al., 2018). 4-AP blocks transient K^+^ currents, prolonging action potentials by inhibiting repolarization. As this can act on both glutamatergic and GABAergic neurons, the release of GABA is reduced, leading to excitation (Avoli, 2014).

GABA antagonism is a major mechanism of seizurogenesis, as the inhibition of GABA’s inhibitory mode of action results in increased excitation (Olsen et al., 1999). A variety of GABA_A_ receptor antagonists can be chosen to cause SLE. Commonly used are: gabazine, picrotoxin, bicuculline, and pentylenetetrazole (PTZ; Easter et al., 2007; Hongo et al., 2015; Bradley et al., 2018). These agents have convulsant effects, with their mechanisms not entirely understood. In the case of bicuculline, for example, its ability to generate SLE comes from preventing Cl^-^ influx, which would normally result in hyperpolarization. Epileptiform activity in iPSC-derived cultures has been observed following treatment with bicuculline (Odawara et al., 2014, 2016b). Although mainly used to induce SLE *in vivo*, PTZ can also be used *in vitro* (Easter et al., 2009) and can induce epileptiform burst firing in iPSC-derived cultures (Odawara et al., 2016b). Picrotoxin has also been used in iPSC-derived neural cultures to assess network activity (Kuijlaars et al., 2016) and Gabazine to generate epileptiform activity (Ishii et al., 2017).

## Future Directions

The production of a robust, efficient, high-throughput human assay for seizure-liability testing is an important contribution to pre-clinical safety studies. While the progress in utilizing new platforms such as iPSC-derived neural cultures for toxicity testing has been impressive, there remain a number of hurdles that still need to be overcome. Notably, the intricacy of the nervous system cannot be completely modeled using only individual cell types. The inclusion of multiple neuronal subtypes including excitatory and inhibitory neurons, astrocytes, oligodendrocytes, and microglia will add further complexity and relevance to model systems.

### Reproducible Generation of Functionally Mature Neurons

While human stem cell derived neurons can be routinely produced using well established methods (Chambers et al., 2009; Shi et al., 2012b) the reproducibility of these methods is variable (Hu et al., 2010). The neurons produced are often slow (2–3 months) to exhibit functional properties such as sustained action potential firing and synaptic plasticity. This represents a significant limitation in experimental models and screening platforms. Alternative approaches such as transdifferentiation allow the direct neuronal cell reprogramming to generate different neuronal lineages, termed “induced neurons” (iN; Vierbuchen et al., 2010) or “induced astrocytes” (iA; Caiazzo et al., 2015). iN can be generated within 3–5 weeks after reprogramming and demonstrate physiological action potential firing (Vierbuchen et al., 2010). Furthermore, iA can be produced within 2 weeks (Stasiukyniene et al., 2009; Caiazzo et al., 2015). While these approaches have reduced the time required to generate functional neuronal subtypes, the efficiency of generating iN cells is often less than 10% (Yang et al., 2011).

### 2D vs 3D Culture

**FIGURE 3:**
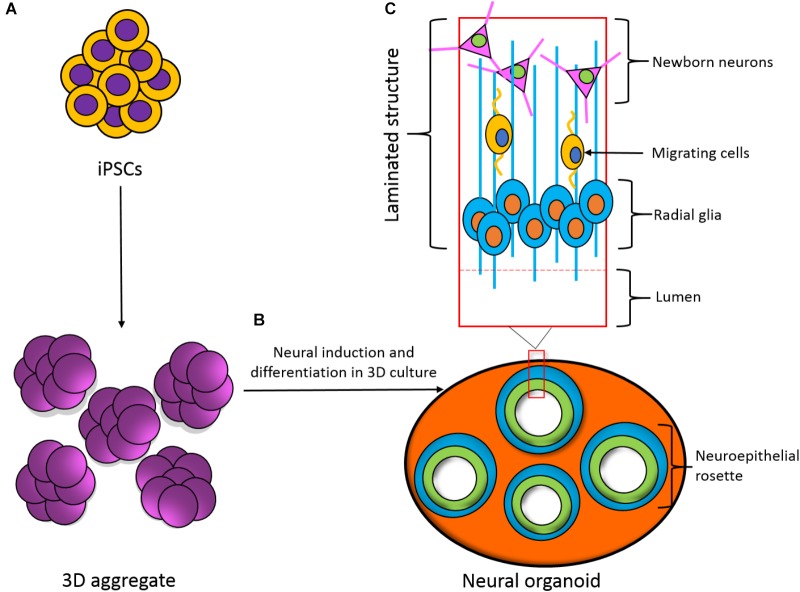
Development of a three-dimensional (3D) neural organoid from IPSCs. **(A)** IPSCs can be spontaneously differentiated within 3D aggregates. **(B)** 3D aggregates can be further cultured in 3D to develop a neural organoid. These neural organoids recapitulate the developmental processes and structural hierarchy seen in the developing brain. **(C)** Section of the laminated structure formed within the neural organoid.

A criticism of the use of two-dimensional (2D) human cultures is that they do not reproduce the structure and hierarchical connectivity that is seen in three-dimensional (3D) tissue. An important route to obtain better structural and morphological relevance is to generate iPSC-derived 3D co-cultures such as organoids and spheroids (**Figure 3**), which have been shown to recapitulate early development of the human cortex (Lancaster et al., 2013; Lancaster and Knoblich, 2014a,b; Pasca et al., 2015). While these 3D cultures are useful models for early development and diseased states, they are less able to model complex, later stages of development and lack vasculature (Sun et al., 2018). Overcoming these issues and applying this technology to seizure-liability testing could provide an insight into not only the mechanisms of seizure spread between layers of cortical cells, but possibly identify novel targets and pharmaceuticals. The advent of matured 3D structures would aim to generate layers of the cortex as seen *in vivo* and provide a robust and relevant platform that resembles a human cortex, in terms of both structure and functionality, adding an extra dimension and increased relevance for human seizure-liability testing.

### Stability of Cultures and Long-Term Recording

Current protocols for generating iPSC-derived neural cultures demonstrate variability in the emergence of functional maturity and cellular composition. Variability between iPSC lines from different patients, as well as variability between closes from the same patient, also represents a significant hurdle in the deployment of *in vitro* screening platforms (Cahan and Daley, 2013). Furthermore, the diversity of differentiation strategies leads to differences in the ratio of cell types, such as neurons and astrocytes, and raises questions over the validity of models in representing human brain tissue. As such, standardization and validation of differentiation protocols for specific applications such as safety pharmacology testing should be considered.

Induced pluripotent stem cell-derived neurons represent a fetal stage (Patani et al., 2012); hence, strategies to enhance the functional maturity of cultures are under investigation (Bardy et al., 2015). However, the ability to monitor the functional maturation of neuronal networks in real time is an important issue in the development of relevant model systems. While methods such as MEA analysis can be used to non-invasively monitor epileptiform phenomena, these systems offer relatively low spatial resolution. However, optical imaging and stimulation techniques can be employed to interrogate neuronal networks at single-cell resolution (Afshar Saber et al., 2018) and offer the opportunity to develop non-invasive, high-throughput screening tools for monitoring seizure liability.

## Conclusion

Over the past 20 years, the application of human iPSCs in drug discovery and safety pharmacology has dramatically increased. The increasing capabilities of iPSC-derived cell culture (in both 2D and 3D) and high-throughput electrophysiological techniques will hopefully provide researchers with the ability to generate functional cell networks which are suited to multiple safety pharmacology applications. In order to be validated and accepted, PSL testing using human iPSCs needs to satisfy the basic requirements for a predictive model: they must express expected human cell types and receptors, they must respond pharmacologically to a wide range of compounds and conditions, and they must display functionality at the single-cell and network level. Meeting all of these criteria would lay the foundation for accepted and robust methods in human drug discovery, which is not only relevant to human systems, but also cost-effective and ethically acceptable.

## Author Contributions

EH conceived the presented idea. AG wrote the manuscript with support from DN, MK, HP, and MC. MK prepared the figures. All authors contributed to the manuscript revision, and read and approved the submitted version.

## Conflict of Interest Statement

The authors declare that the research was conducted in the absence of any commercial or financial relationships that could be construed as a potential conflict of interest.
